# The Influence of Manganese Slag on the Properties of Ultra-High-Performance Concrete

**DOI:** 10.3390/ma17020497

**Published:** 2024-01-20

**Authors:** Wenyu Xu, Jia Yu, Hui Wang

**Affiliations:** 1 School of Intelligent Manufacturing, Nanjing University of Science and Technology Zijin College, Nanjing 210023, China; xuwenyu@aliyu.com (W.X.); yujia@aliyun.com (J.Y.); 2 School of Civil Engineering and Geographic Environment, Ningbo University, Ningbo 315000, China

**Keywords:** manganese slag, ultra-high-performance concrete, plastic viscosity, energy spectrum analysis, X-ray diffraction

## Abstract

Manganese slag (MS) is a kind of chemical waste, which may pollute the environment if conventional handling methods (stacking and landfill) are applied. Ultra-high-performance concrete (UHPC)—with considerably high compactness and strength—can be used not only as a special concrete material, but also to solidify the toxic substances in solid waste. This study proposes the addition of MS to UHPC, where the mass ratio of MS varies from 0% to 40% in the total mass of MS and silica fume. The effects of MS on the fluidity, plastic viscosity, and yield shear stress are investigated, and the flexural strength, compressive strength, and dry shrinkage rate of UHPC with MS are measured. X-ray diffraction (XRD) spectrum and energy spectrum analysis (EDS) diagrams are obtained to analyze the performance mechanism of the UHPC. A rheological study confirms that the slump flow increases with the increasing rate of 0–14.3%, while the yield shear stress and plastic viscosity decrease with the rates of 0–29.6% and 0–22.2%, respectively. The initial setting time increases with the mass ratio of MS by 0–14.3%, and MS has a positive effect on the flexural and compressive strengths of UHPC. In the early curing stage (less than 14 days), the increasing rate in the specimens increases with the curing age; meanwhile, when the curing age reaches 14 days or higher, the increasing rate decreases with increasing curing age. The compactness of UHPC is increased by adding MS. Furthermore, MS can increase the elements of Al and decrease crystals of Ca(OH)_2_ and calcium silicate hydrate in UHPC.

## 1. Introduction

Manganese slag (MS) is a strategic resource widely used in industries such as metallurgy and the chemical industry [1,2]. According to statistics, the accumulation of electrolytic manganese slag due to historical and technical issues may have exceeded 100 million tons, with an annual increase of 10 million tons [3,4,5]. At present, the resource utilization and comprehensive utilization methods for electrolytic MS are not mature, and there are few large-scale applications [6,7,8,9]. The new round of environmental policies has put forward higher requirements for the comprehensive treatment of electrolytic MS, and the increase in environmental investments has significantly increased production costs [10,11]. Therefore, it is imperative to promote the comprehensive utilization and reduction of electrolytic MS, and research on environmentally friendly resource utilization technologies is urgently needed. If MS is not treated in a timely and reasonable manner, it can pollute water sources. At the same time, the metal elements in manganese slag will circulate to the human body, thereby endangering human health and safety [12,13].

At present, stacking and landfill methods are used for the treatment of MS [14,15]. Direct landfilling of electrolytic MS is a simple and convenient method [16,17]; however, long-term open-air storage of electrolytic MS not only occupies a large amount of land, but also accumulates pollutants from the long-term storage of electrolytic MS in the landfill site into leachate waste water under the action of rainfall [18]. High concentrations of pollutants can accumulate in the leachate, posing a significant potential risk of pollution [19]. Based on these reasons, MS needs to be permanently solidified.

MS has been shown to improve the mechanical strength of cement-based materials. Due to the large amount of active substances in MS, crushed and ground MS not only has a microaggregate filling effect on the cement but, also, can increase the hydration process [20,21]. In this way, the pore structure inside the concrete is improved, thus increasing the mechanical strength of cement concrete [22]. Maurilio et al. have reported that the addition of MS decreased the porosity of cement concrete cured for 28 days, thus increasing the corresponding compressive strength at a rate of 0–42.1%. Meanwhile, the flexural strength was increased by the maximum rate of 41.7% [23]. Moreover, MS has been proved to improve the chloride ion penetration resistance and frost resistance of concrete [24]. However, ordinary concrete possesses a large number of pores; therefore, the toxic substances may leach from the cement concrete.

Ultra-high-performance concrete (UHPC) is a cement concrete with excellent mechanical strength and durability. As obtained from Zhang’s researches, UHPC with reinforced fibers showing high toughness can strengthen RC beams in torsion [25]. However, UHPC presents quite high drying shrinkage, leading to severe cracking [26]. As reported in prior researches, the dry shrinkage rate of UHPC has been applied in characterizing its cracking performance [27]. Yalçınkaya et al. have proved that the addition of fly ash, silica fume, blast furnace slag powder, etc., can reduce the shrinkage and cracking of UHPC by the dry shrinkage value testing method and the ultrasonic testing method [28,29]. Yoo et al. have determined that UHPC with fibers can be used as twisted reinforced concrete beams. Some chemical wastes (e.g., waste fly ash, secondary aluminum ash, and river silt) can be used for the preparation of UHPC. Waste fly ash and secondary aluminum ash increased the flexural strengths of UHPC by rates of 0–23.6% and 0–27.1%, respectively. Meanwhile, the corresponding compressive strengths were improved by 0–18.6% and 0–21.7% [30]. Moreover, the recycled aggregate concrete and UHPC’s resistance to high temperature can be improved by adding the waste rubber and the plant fibers. Furthermore, the natural fibers with low cost can be used to prevent the reinforced concrete’s corrosion. However, the effect of natural fibers on cement concrete’s mechanical strengths are limited [31,32,33,34]. Therefore, the natural fibers are not suitable as reinforcing materials for UHPC. At the same time, the high compactness can prevent the toxic substances from leaching. Therefore, UHPC with such excellent mechanical, durability, and compactness can be used to solidify the MS. However, this issue has received little attention in the literature.

The principal aim of this study is to assess the influence of MS on the slump flow, plastic viscosity, yield shear stress, and initial setting time of UHPC. The mechanical strengths and the drying shrinkage rates of UHPC cured for 1 day, 3 days, 7 days, 14 days, and 28 days were measured, and scanning electron microscope imagery, energy spectrum analysis results, and X-ray diffraction spectra were obtained to analyze the inner performance mechanism of the UHPC. This study provides a new approach for the treatment of MS.

## 2. Materials and Methods

### 2.1. Raw Materials

**Figure 1:**
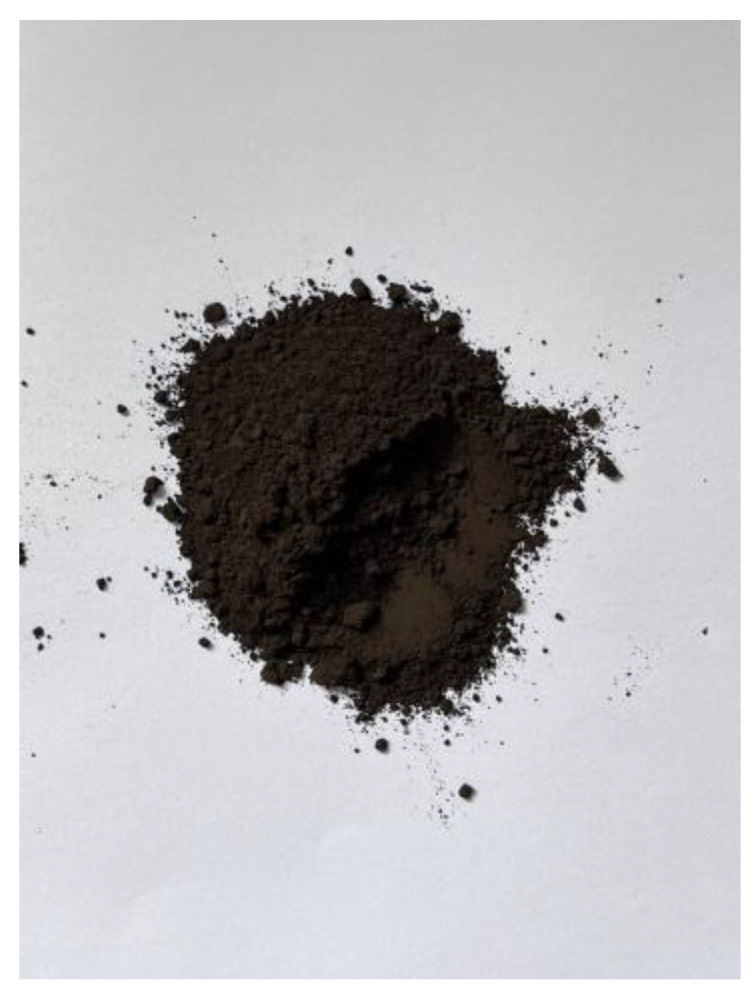
The manganese slag used in the UHPC.

The Ordinary Portland cement (OPC) used in the current work was procured from the Jiangsu Huaxi Cement Manufacturing Co., Ltd. in Wuxi, China. The cement has a density of 3.2 g/cm^3^, and the initial and final setting times were 121 min and 233 min, respectively. Ultra-fine silica fume (SF) showing a density of 2.33 g/cm^3^, specific surface area of 14.82 m^2^/g, and 98.1% SiO_2_ was applied in this study as a supplementary cementitious material. Shandong Zhongfen Building Materials Technology Co., Ltd., Jinan, China, provided the blast furnace slag powder (BFP), which has a density of 2.79 g/cm^3^, specific surface area of 433.7 m^2^/g, and loss on ignition of 2.18%. Quartz sand (QS) with three kinds of particle size (1–0.5 mm, 0.5–0.1 mm, and 0.1–0.01 mm), sourced from Guangzhou Yifeng Chemical Technology Co., Ltd. of Guangzhou, China, was used as the aggregate. The fluidity of the fresh UHPC was adjusted using an efficient polycarboxylic acid water reducing agent provided by Henan Jinrong Cement Co., Ltd. in Sanmenxia, China. The manganese slag (MS) shown in Figure 1 was purchased from the Hunan Daji Manganese Industry Co., Ltd., Hengyang, China, which was used for testing and measurement in this study.

**Figure 2:**
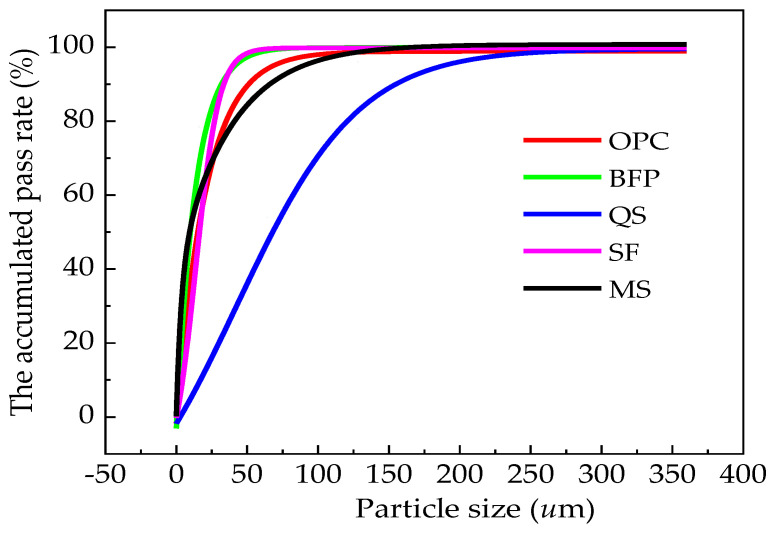
The particle size distribution curves of the raw materials.

Table 1 and Table 2 detail the accumulated pass rates and chemical compositions of the raw materials. The particle size distribution curves of the raw materials are shown in Figure 2.

### 2.2. Preparation of the Specimens

Table 3 provides the UHPC mixing ratios. The UHPC specimens were fabricated by the following steps. The powder binder materials were mixed in a JJ-5 planetary cement mortar mixer from Wuxi Jianyi Instrument Machinery Co., Ltd., Wuxi city, China with a stirring speed of 140 rpm for 30 s. Then, the quartz sand was added with stirring at 285 rpm for 90 s. Finally, water mixed with the water reducing agent was added to the mixture and stirred at a speed of 285 rpm for 120 s.

### 2.3. Measuring Methods

#### 2.3.1. The Measurement of Rheological Parameters

**Figure 3:**
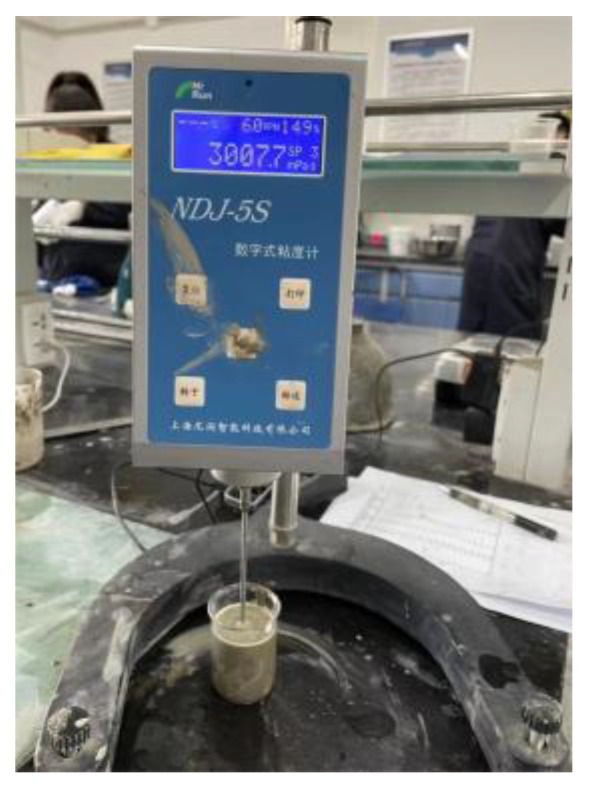
The measurement of plastic viscosity and yield shear stress.

An NDJ-5S Rheometer with a mixing speed of 0–30 rpm was used for measurement of the plastic viscosity and yield shear stress of fresh UHPC. The slump flow was tested using the jumping table method. The study in [35] provides the detailed measuring process of the rheological parameters. Figure 3 shows the measurement of plastic viscosity and yield shear stress.

#### 2.3.2. The Initial Setting Time

**Figure 4:**
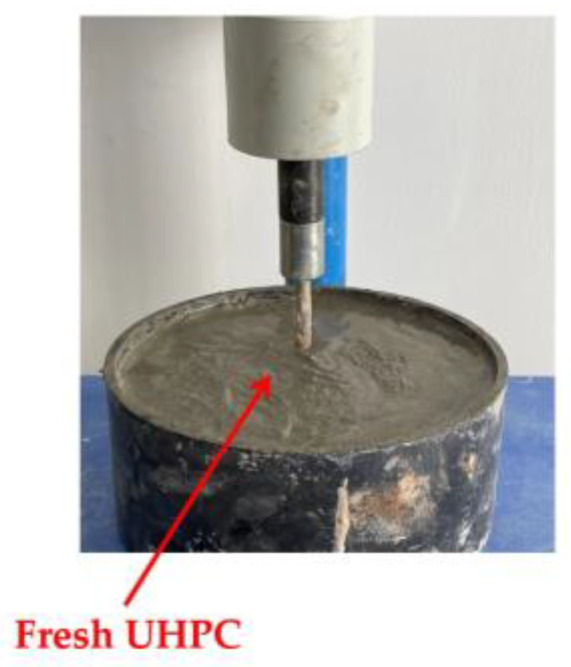
The measurement of initial setting time.

A digital display mortar setting time tester (Cangzhou Qiuzhen Instrument Equipment Co., Ltd., Cangzhou, China) was used to measure the initial setting time of fresh UHPC. The measuring process for the initial setting time of fresh UHPC is shown in Figure 4.

#### 2.3.3. The Mechanical Strengths

**Figure 5:**
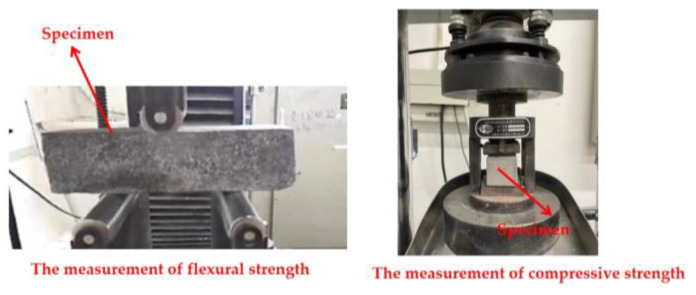
The measuring process of UHPC’s mechanical strengths.

The flexural and compressive strengths were measured using a fully automatic bending integrated testing machine. The mechanical strength tests were conducted using 40 × 40 × 160 mm^3^ specimens at loading rates of 0.1 kN/s and 2.4 kN/s, respectively. The mechanical strength measurement processes for UHPC are shown in Figure 5.

Samples were utilized to determine the toxic heavy metal substances released during 6 months of immersion in deionized water. Monthly measurements of C_r_ and Z_n_ in immersion were carried out using an Inductively Coupled Plasma Emission Spectrometer (Suzhou Huapu Scientific Instrument Co., Ltd., Suzhou, China).

An SU3800 scanning electron microscope, purchased from Hitachi Scientific Instruments (Beijing) Co., Ltd., Beijing, China was applied to acquire the SEM images and the EDS results. At first, all of the samples were removed from the core of the specimens. Then, they were dried in an oven at a temperature of 105 °C for 2 days and coated by vacuum gold spraying. Finally, the sprayed samples were transferred to the SU3800 for SEM and EDS measurements.

The powdered samples were analyzed using a Bruker JV-DX X-ray diffractometer from Shanghai Erdi Instrument Technology Co., Ltd. of Shanghai, China, and the resulting X-ray diffraction curves were obtained. Additionally, a portion of the powder was analyzed by thermogravimetric analysis utilizing a TGA thermogravimetric analyzer provided by Estone Technology (Hong Kong) Co., Ltd., Hong Kong, China.

## 3. Results and Discussion

### 3.1. The Rheological Parameters of UHPC

**Figure 6:**
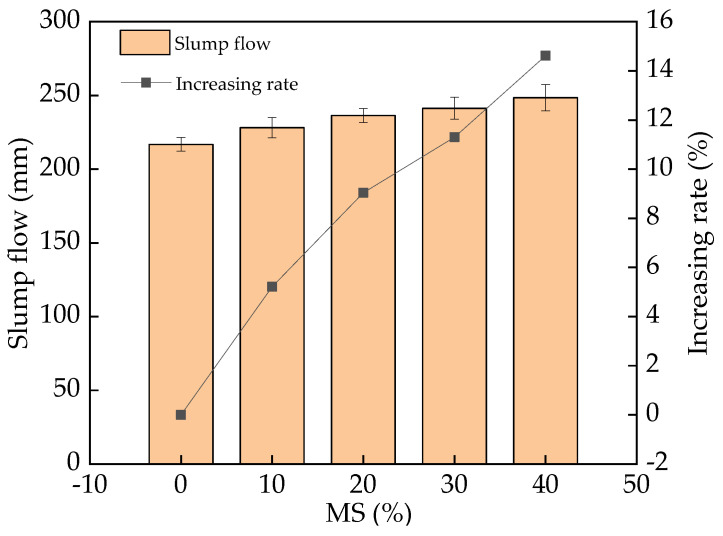
The slump flow of UHPC with MS.

Figure 6 displays the slump flow of the fresh UHPC. It was found that the slump flow presents an upward trend with increasing MS dosage. The increasing rate of slump flow with MS varied from 0% to 14.3%. The flowability of fresh UHPC is decided by its inner free water and the shapes of the used materials [24]. MS has smaller specific surface area than SF, leading to less adsorption of free water and higher slump flow. Furthermore, it can be noted that MS has a more rounded micromorphology than cement and SF [36]. MS, with its rounded shape, can reduce the flow resistance in fresh UHPC, thus increasing its slump flow. The ball effect of MS can possibly increase the fluidity of fresh UHPC. The error bar values of fresh UHPC’s slump flow were lower than 8% of the slump flow values, confirming the accuracy of the experiment.

**Figure 7:**
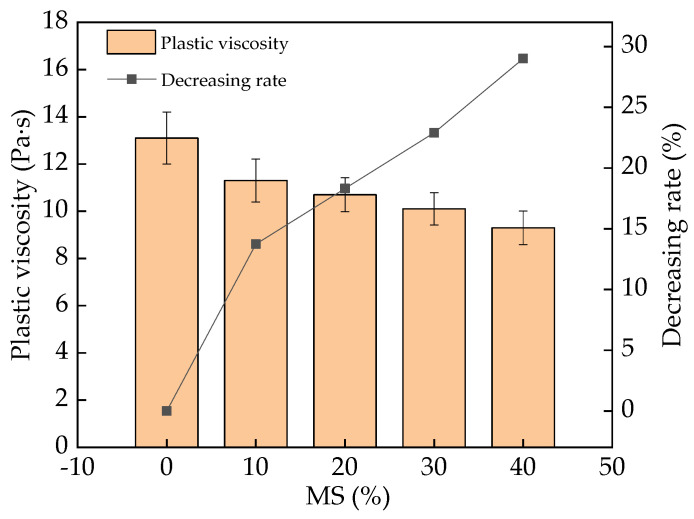
The plastic viscosity of UHPC with MS.

The plastic viscosity of fresh UHPC is shown in Figure 7. Contrary to the research results on slump flow, the plastic viscosity presented a downward trend, with decreasing rates of 0–29.6%, with the addition of MS. As has been pointed out in prior studies [37,38], the relationship between the plastic viscosity and the slump flow shows an inverse correlation. Therefore, an increased slump flow leads to the decreased plastic viscosity. On one hand, the MS combines less free water, leading to the increased fluidity in fresh UHPC and, thus, the plastic viscosity is reduced by adding MS. On the other hand, the ball effect of MS can increase the slump flow of fresh UHPC, as analyzed above, thus decreasing its plastic viscosity [39]. The error bar values of plastic viscosity for fresh UHPC were lower than 7.6%, ensuring the experimental accuracy.

**Figure 8:**
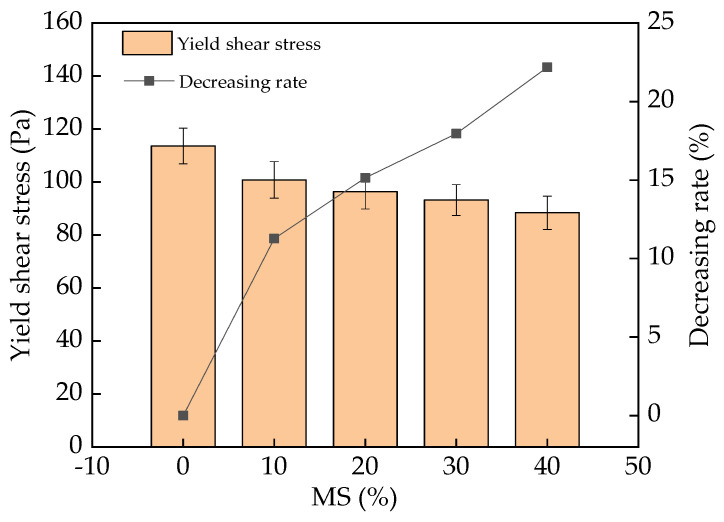
The yield shear stress of UHPC with MS.

The yield shear stress of fresh UHPC with different dosages of MS is depicted in Figure 8. As expected, the variation law of yield shear stress was the same as that for plastic viscosity. Previous studies have pointed out that, at the same shear rate, the yield shear stress of the fresh paste is positively correlated with the plastic viscosity [40]. Therefore, the yield shear stress of fresh UHPC showed a similar trend as plastic viscosity. The decreasing rates of the yield shear stress were 0–23.1%. The specific reasons for this variation were elaborated earlier. The corresponding error bar values were lower than 7.3% of the yield shear stress values, guaranteeing the precision of the experimental results.

### 3.2. The Initial Setting Time of UHPC

**Figure 9:**
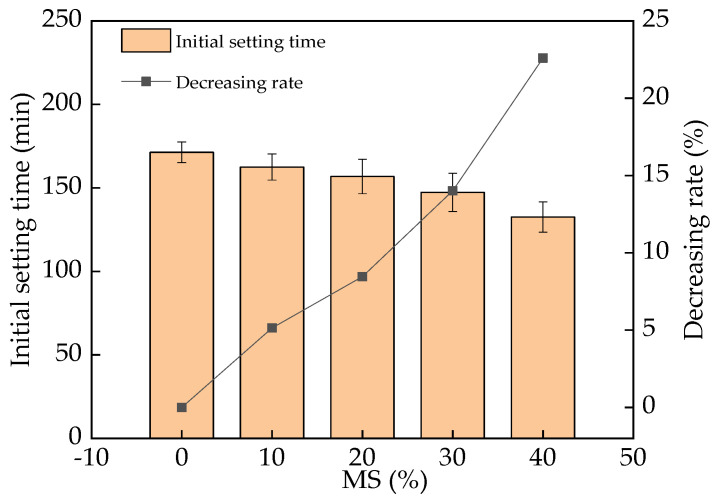
The initial setting time of UHPC with MS.

The initial setting time of UHPC is shown in Figure 9. From Figure 9, the initial setting time shows a reducing trend with the mass ratio of MS. As has been previously reported, the hydration rate of hydraulic substances (e.g., Al_2_O_3_) in MS is higher than that of hydraulic substances in cement [41,42,43]. Consequently, the addition of MS led to an increase in the early hydration rate of cement and a reduction in the initial setting time. The decreasing rates of initial setting time with the addition of MS were 0–23.1%. The error bar values were lower than 8.1% with respect to the real initial setting time values, indicating the accuracy of experimental results.

### 3.3. The DSR of UHPC

**Figure 10:**
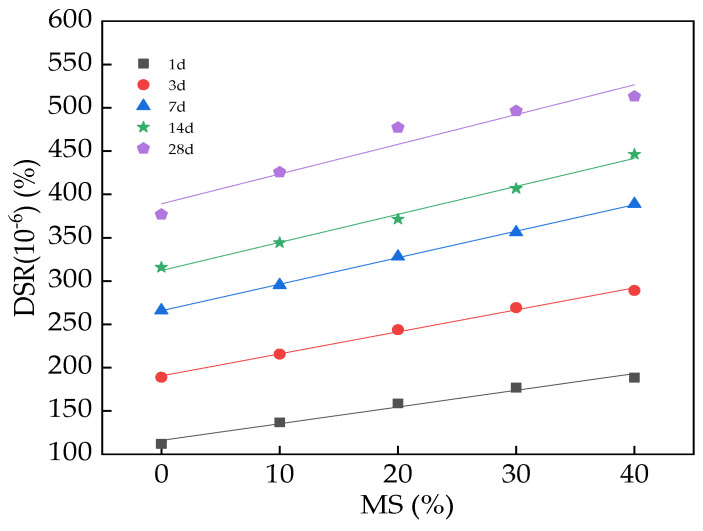
The DSR of UHPC with MS.

The results for the DSR are given in Figure 10, which indicates that the DSR of UHPC increases in a linear manner with the mass ratio of MS and increasing curing age. Table 4 shows the fitting results between the DSR and the mass ratio (M) of MS. The increasing rates of DSR varied from 33.1% to 42.3% when adding MS. This is mainly due to the fact that the hydration activity of MS is higher than that of the cement, resulting in a faster hydration rate of MS [44,45]. Therefore, the drying shrinkage rate increased with increasing MS content. The increasing rates of the DSR were 0–213.2% and 0–31.2% with the curing age and the addition of MS. Evidently, the hydration degree increases with the curing age, which leads to an increased drying shrinkage rate. It can be observed, from Figure 10, that the error bar values were lower than 6.7% of the DSR values, demonstrating the accuracy of the research results. The fitting degrees of the equations were all higher than or equal to 0.92, ensuring the rationality of the fitting results. 

### 3.4. The Mechanical Strength of UHPC

**Figure 11:**
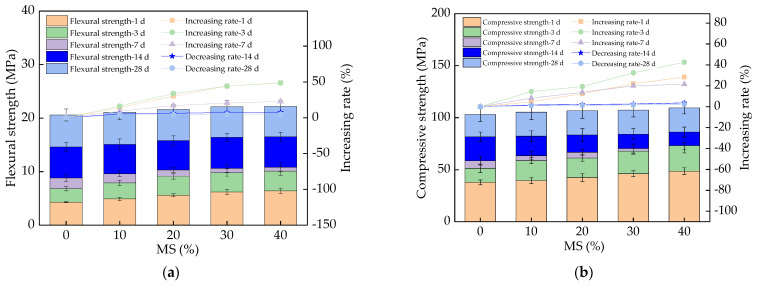
The mechanical strengths of UHPC with MS. (**a**) The flexural strength; (**b**) The compressive strength.

The flexural and compressive strengths of UHPC with MS are displayed in Figure 11, from which it can be seen that the flexural strengths of UHPC increased, with increasing rates of 0–28.3%, 0–48.2%, and 0–21.5%, when the curing age was 1 day, 3 days, and 7 days, respectively. This can be explained by the substances having higher early hydration activity, improving the flexural strength in the early curing stage (i.e., less than 14 days) [46,47]. However, when the curing age was 14 days and 28 days, the flexural strengths of UHPC increased with the addition of MS, with the increasing rates of 0–2.8% and 0–2.1%, respectively. This can be attributed to the fact that the MS can increase the early hydration rate and hydration heat, thus increasing the cracks in UHPC and decreasing the increasing rate of the flexural strength. Figure 11 indicates that the compressive strengths varied with the same changing rules as the flexural strengths. In the early curing stage (lower than 14 days, i.e., 1, 3, and 7 days), the increasing rates of compressive strengths with MS were 0–26.1%, 0–42.5%, and 0–20.2%, respectively. When the curing ages were 14 days and 28 days, the increasing rates of compressive strengths with MS were 0–3.6% and 0–2.2%, respectively. When compressive loading was applied to the specimens, the direction of vertical compression caused tensile failure; thus, the essence of UHPC’s compression failure is tensile failure. Therefore, the mechanism of its compressive strength change is the same as that of its flexural strength described above. Hence, the compressive strength was improved obviously through the addition of MS, due to the obviously increased early hydration rate [48]. However, the compressive strengths increased insignificantly, which can be attributed to the increased cracks due to the increased early hydration heat [49]. Apparently, the flexural and compressive strengths increased with the increasing rates of 0–316% and 0–223% with an increase in the curing age, ranging from 1 day to 28 days. The error bars of mechanical strengths were lower than 7.3% of the mechanical strength values, indicating the accuracy of the experimental results.

### 3.5. The Electrical Resistance of UHPC

**Figure 12:**
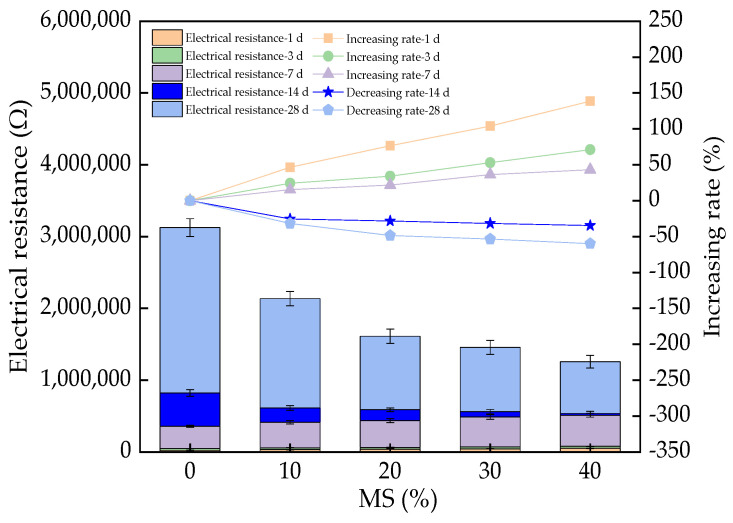
The electrical resistance of UHPC with MS.

The electrical resistance of UHPC is illustrated in Figure 12, which shows that the electrical resistance increased with increasing rates of 0–138.6%, 0–71.1%, and 0–43.2%, respectively, with MS dosages of 0–40% when the curing age was lower than 14 days. As the hydration activity of UHPC with MS is higher than that of cement, the UHPC with MS can consume more water, leading to a decrease in the concentration of the pore solution [50]. Electrical conduction is mainly determined by the electrical resistance of the pore solution [51]. As a result, the electrical conduction was decreased by MS. Hence, the electrical resistance of the UHPC was increased. However, when the curing age reached 14 days, the electrical resistance was decreased by MS, with decreasing rates of 0–34.6% and 0–59.8%, due to the increased conductive effect of manganese [52]. Moreover, the electrical resistance of UHPC increased with the curing age if the MS was maintained at the same dosage. This can be explained by the decreased pore solution due to the effect of curing age on the hydration rate [53].

**Figure 13:**
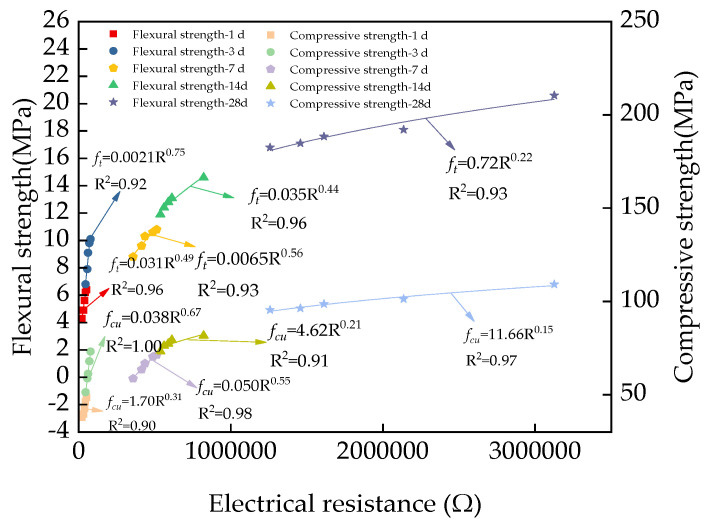
The fitting equations between the electrical resistance and the mechanical strengths of UHPC with MS.

The fitting equations between the mechanical strengths and the electrical resistance are shown in Figure 13. It can be observed, from Figure 13, that the relationships between the mechanical strengths and the electrical resistances coincided with power functions. The fitting degrees of all fitting equations were no lower than 0.90, which confirms the accuracy of the experimental results. This can be explained by the fact that the electrical resistance depends on the pore solution of UHPC [54]. Meanwhile, the mechanical strengths of UHPC were closely related to the electrical resistance. Therefore, the electrical resistance of UHPC has a particular relationship to its mechanical strength. Consequently, the electrical resistance of UHPC can be used to calculate its mechanical strength.

### 3.6. The AC Impedance Spectrum of UHPC

**Figure 14:**
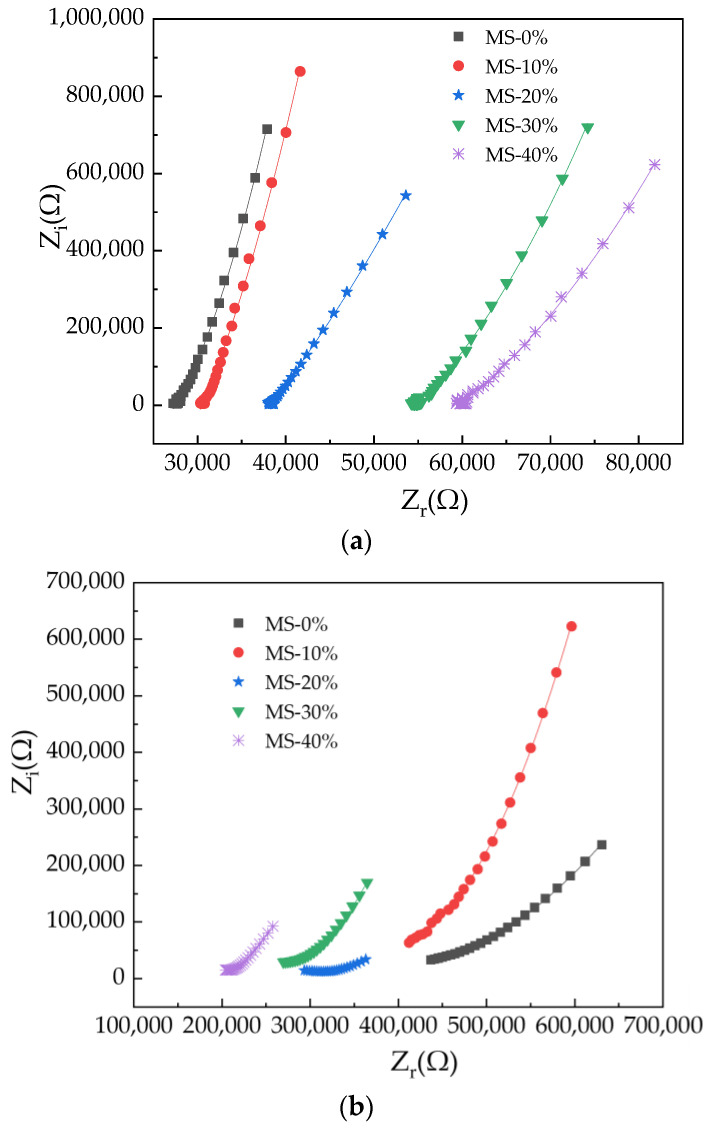
The AC impedance spectrum of UHPC with MS. (**a**) UHPC with MS cured for 3 days; (**b**) UHPC with MS cured for 14 days.

The AC impedance spectrum of UHPC is shown in Figure 14. In Figure 14, Z_r_ and Z_i_ stand for the electrical resistance and electrical reactance, respectively. The fitting results are shown in Table 5, from which it can be observed that the electrical reactance of the UHPC varied in the form of a quadratic function, with respect to its electrical resistance. The fitting degrees were all 1.0, demonstrating the accuracy of the fitting results. This can be explained by the interfaces between different solids and the interfaces of solids and liquids [55]. The interfaces of different phases induce variation in the electrical reactance. As described above, the electrical resistance of UHPC is dominated by the electrical conduction of the inner pore solution. As a result, a specific relationship exists between the resistance and capacitance of UHPC [56]. It can be observed, from Figure 14, that the electrical resistance value corresponding to the extreme points increases with the curing age. This electrical resistance value first increases with the addition of MS (curing age less than 14 days); however, when the curing age reaches 14 days, the electrical resistance value is decreased with the addition of MS. The variation was analyzed according to the results of the AC electrical resistance above.

**Figure 15:**
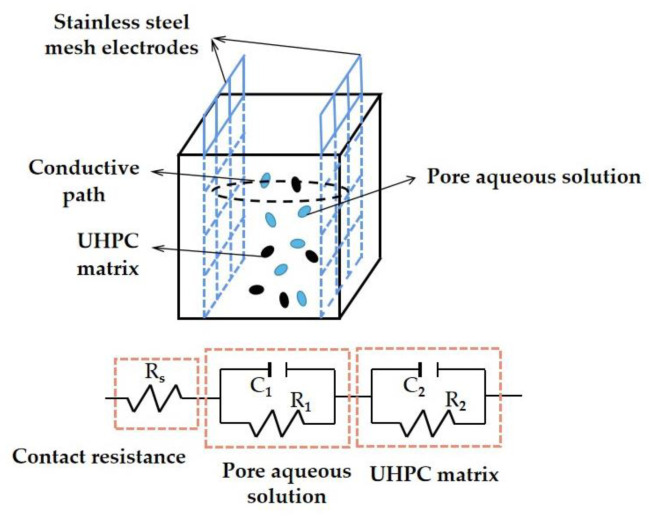
The equivalent circuit of UHPC with MS.

The equivalent circuits of UHPC are shown in Figure 15. It can be seen that the circuit diagram for UHPC consists of four sets of electrical components. The three sets of electrical components are introduced as follows. The parallel electrical resistances and reactances of the pore solution and the UHPC matrix are connected in series, while the third electrical component is the contact electrical resistance of the stainless steel electrode meshes between the UHPC matrix. The Chi values of the UHPC equivalent circuits were no higher than 0.026, indicating the accuracy of the equivalent circuit diagrams.

### 3.7. The Leached Toxic Substances

**Figure 16:**
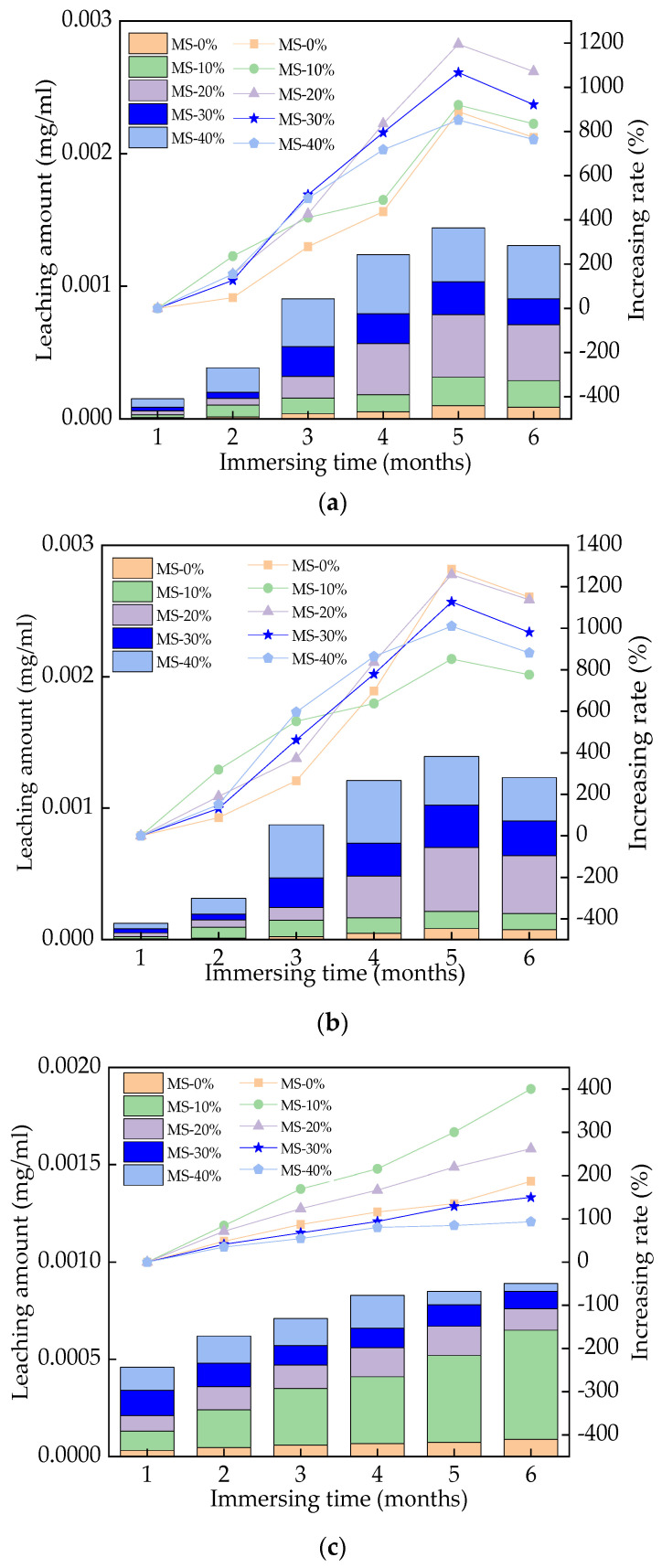
The leached toxic elements. (**a**) The leaching C_r_ of UHPC with MS; (**b**) The leaching Z_n_ of UHPC with MS; (**c**) The leaching M_n_ of UHPC with MS.

The leached Cr, Zn, and Mn from UHPC are shown in Figure 16, from which it can be seen that the leached Cr, Zn, and Mn increased with increasing immersion time and the dosage of MS. The increasing rates of Cr, Zn_,_ and Mn leached from UHPC were 0–1218%, 0–1312.3%, and 0–406.8%, respectively, with respect to the immersion time. Meanwhile, the increasing amounts of Cr, Zn, and Mn in UHPC were 0–0.0361 mg/mL, 0–0.0353 mg/mL, and 0–0.00317 mg/mL, respectively, with respect to the dosage of MS. MS, having a certain amount of toxicity, can cause some environmental pollution. Compared with other studies, the leached Cr, Zn, and Mn from the UHPC tested in this study were much lower than the toxic elements leached from ordinary cement concrete [57,58]. Therefore, the toxic elements seeping out of UHPC have minimized environmental pollution.

### 3.8. The SEM-EDS of UHPC

**Figure 17:**
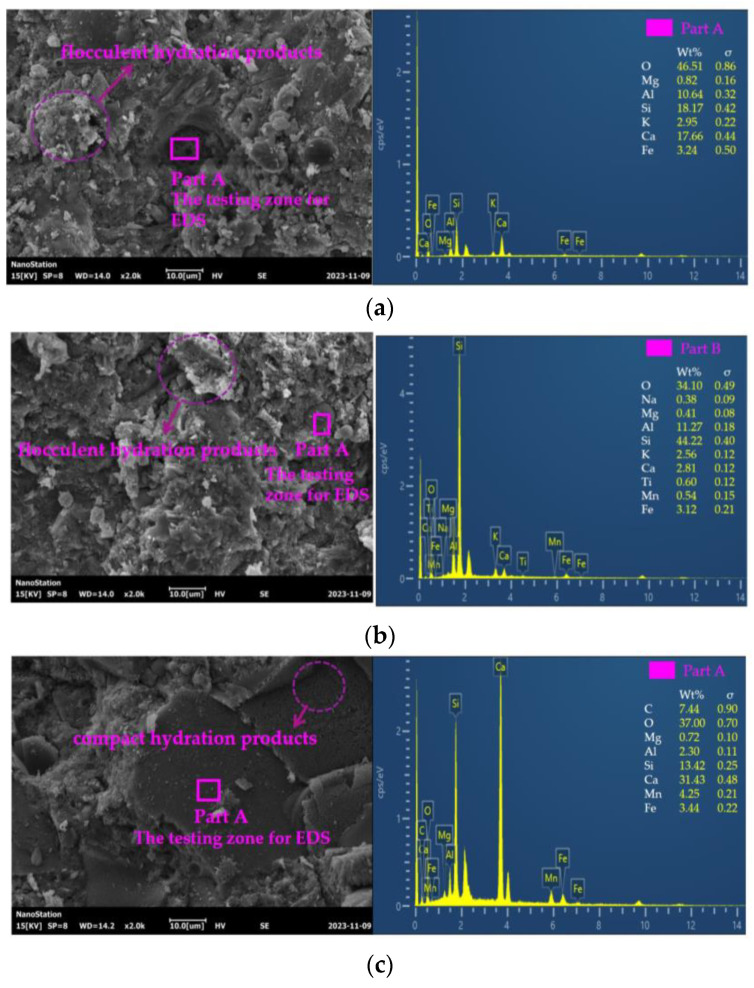
The EDS of UHPC. (**a**) UHPC with 0% MS; (**b**) UHPC with 0% MS; (**c**) UHPC with 40% MS.

The SEM-EDS of UHPC is depicted in Figure 17. It can be observed, from Figure 17, that flocculent hydration and compact products were found in the UHPC. When the dosage of MS increased, the compact hydration products also increased. The elements O, Mg, Al, Si, K, Ca, and Fe were observed in every sample. When MS was added, the elements Na, Ti, and Mn were additionally discovered. The element Mn increased with the addition of MS. The addition of MS can improve the compactness of the UHPC, thus increasing the mechanical strengths of UHPC.

### 3.9. The XRD of UHPC

**Figure 18:**
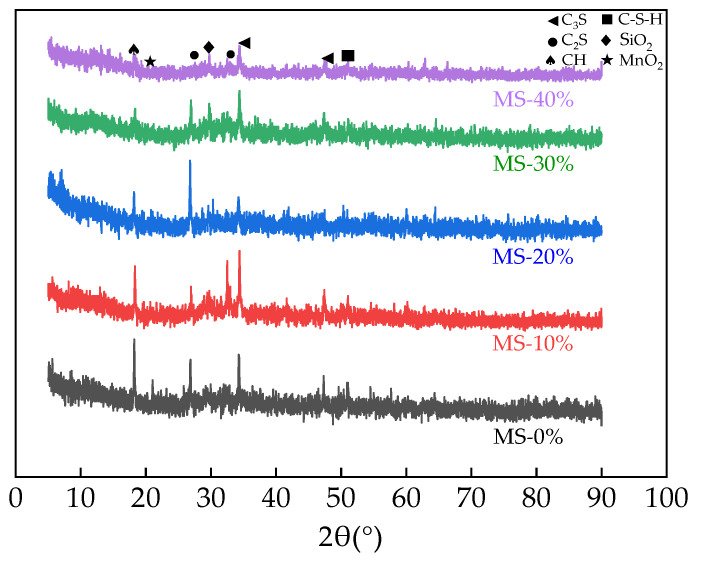
The XRD curves of UHPC.

XRD curves of the UHPC are exhibited in Figure 18. From Figure 18, the UHPC consisted of Ca(OH)_2_ (CH), 3CaO·SiO_2_, SiO_2_, calcium silicate hydrate (C-S-H) crystals, and MnO_2_. When the amount of MS increased, the C-S-H crystals increased and the CH crystals decreased. This can be explained by the increased pozzolanic effect of MS in UHPC [59]. It can be observed, from the XRD curves, that the diffraction peak of MnO_2_ was not obvious, confirming that the MnO_2_ had been solidified into a cement hydration substance in UHPC. Hence, M_n_ can be effectively disposed through its addition into UHPC.

## 4. Conclusions

The conclusions of this research, obtained from the above results, can be summarized as follows.

The addition of MS (0–40% in the mass ratio of the total binder materials) increased the slump flow of fresh UHPC, with increasing rates of 0–14.3%. The corresponding plastic viscosity and yield shear stress were decreased by 0–29.6% and 0–23.1%, respectively, with the addition of MS.

The initial setting time of UHPC was increased by MS. The DSR of UHPC presented a positive linear relationship with the mass ratio of MS. The increasing rates of DSR were 0–213.2% and 0–31.2% with respect to the curing age and the added MS, respectively.

The flexural and compressive strengths were increased by 0–48.2% and 0–22.5% with the addition of MS when the curing age was lower than 14 days; however, the flexural and compressive strengths were decreased by 0–2.8% and 0–3.6% after the curing age reached 14 days.

The electrical resistance of UHPC was first increased with the addition of MS and then decreased in the curing stages of 1–7 days and 7–28 days, respectively. The electrical resistance presented power function relationships with the flexural and compressive strengths of the UHPC.

The Cr, Zn, and Mn leached from UHPC increased with increasing rates of 0–1218%, 0–1312.3%, and 0–406.8%, respectively, with the mass ratio of MS ranging from 0% to 40% and the immersion time ranging from 1 to 6 months.

MS can increase the compactness of hydration products and increase the Mn elemental composition in UHPC. Furthermore, the Ca(OH)_2_ crystals in the hydration products were decreased with the addition of MS.

## Figures and Tables

**Table 1:** The accumulated pass rate (%).

Types	Particle Size/μm						
	0.3	0.6	1	4	8	64	360
OPC	0.12	0.37	3.09	14.77	29.14	92.61	100
SF	32.04	59.01	82.99	99.8	99.9	100	100
BFP	0.03	0.11	3.4	19.3	35.1	98.3	100
QS	0	0	0	0	0.036	23.95	100
MS	0.05	0.32	1.33	8.72	23.52	98.43	100

**Table 2:** Chemical composition (%).

Types	SiO_2_	Al_2_O_3_	Fe_x_O_y_	MgO	CaO	SO_3_	K_2_O	MnO_2_	Loss on Ignition
OPC	20.2	5.7	3.8	1.8	62.3	2.9	-	-	3.0
SF	90.1	0.3	0.6	0.3	0.45	0.2	7.4	-	-
BFP	33.8	14.7	0.6	9.9	37.1	0.2	3.4	-	-
QS	98.4	-	1.4	-	-	-	-	-	-
MS	34.22	7.32	12.03	1.91	13.92	0.08		30.52	-

**Table 3:** The mixing proportions of UHPC (kg/m^3^).

Types	Water	OPC	MS	SF	BFP	QS	WR
MS-1	240.2	839.9	0	910	111.1	961.5	16.0
MS-2	240.2	839.9	91	819	111.1	961.5	16.0
MS-3	240.2	839.9	182	728	111.1	961.5	16.0
MS-4	240.2	839.9	273	637	111.1	961.5	16.0
MS-5	240.2	839.9	364	546	111.1	961.5	16.0

**Table 4:** The fitting results between the *DSR* and the mass ratio (*M*) of MS.

Equation	Types	*a*	*b*	R^2^
*DSR = a + bM*	DSR(10^−6^)(%)-1d	116	1.93	0.97
	DSR(10^−6^)(%)-3d	190.52	2.54	0.99
	DSR(10^−6^)(%)-7d	265.7	3.06	0.99
	DSR(10^−6^)(%)-14d	312.2	3.24	0.99
	DSR(10^−6^)(%)-28d	389.14	3.43	0.92

**Table 5:** The fitting results of the equations of the Z_i_ and Z_r_.

Equation	Types	A	B	C	R^2^
$Z_{i}=A{Z_{r}}^{2}+BZ_{r}+C$	MS-0%-3 days	0.0030	−123.33	1.16 × 10^6^	1.00
	MS-10%-3 days	0.0021	−75.22	3.08 × 10^5^	1.00
	MS-20%-3 days	3.83 × 10^−4^	0.51	−5.79 × 10^5^	1.00
	MS-30%-3 days	8.19 × 10^−4^	−68.29	1.29 × 10^6^	1.00
	MS-40%-3 days	4.84 × 10^−4^	−40.41	6.89 × 10^5^	1.00
	MS-0%-14 days	3.70 × 10^−6^	−2.88	5.85 × 10^5^	1.00
	MS-10%-14 days	1.29 × 10^−5^	−9.95	1.98 × 10^6^	1.00
	MS-20%-14 days	8.31 × 10^−6^	−5.18	8.19 × 10^5^	1.00
	MS-30%-14 days	1.55 × 10^−5^	−8.34	1.15 × 10^6^	1.00
	MS-40%-14 days	2.23 × 10^−5^	−8.77	8.75 × 10^5^	1.00

## Data Availability

Data is unavailable due to privacy or ethical restrictions.
